# A compressed image encryption algorithm leveraging optimized 3D chaotic maps for secure image communication

**DOI:** 10.1038/s41598-025-95995-8

**Published:** 2025-04-23

**Authors:** Akshat Tiwari, Prachi Diwan, Tarun Dhar Diwan, Mahdal Miroslav, S. P. Samal

**Affiliations:** 1 Department of Computer Science, Kalinga University, Raipur, India; 2 Department of Computer Science , Atal Bihari Vajpayee University, Bilaspur, India; 3 Faculty of Mechanical Engineering, VSB-Technical University of Ostrava, 70800 Ostrava Poruba, Czech Republic; 4 Department of Biosciences, Saveetha School of Engineering, Saveetha Institute of Medical and Technical Sciences, Chennai, 602105 India

**Keywords:** Secure communication, Chaotic maps, Optimization, Compressed encryption, Image encryption, Environmental biotechnology, Plant biotechnology, Environmental sciences, Diseases, Energy science and technology, Engineering, Mathematics and computing

## Abstract

In today’s digital age, sensitive multimedia informations are transmitted over public networks that are vulnerable to unauthorized access and data tampering. This motivates more robust encryption methods to combat such security threats. In this paper, a chaotic map-based encryption technique is presented as a solution to these issues. The proposed algorithm termed as OptiSecure-3D presents optimized parameter-based 3D chaotic maps for image encryption. The method integrates three primary components: stacked autoencoder (SAE), optimized parameter-based chaotic mapping, and encryption/decryption module, to ensure robust and secure encryption of images. The result evaluated the proposed OptiSecure-3D image encryption algorithm with a randomness test, pixel adjacency correlation test, and differential analysis. The mean entropy was approx. 7.9 and the mean number of pixels changing rate (NPCR) was approx. 99.8, unified average changing intensity (UACI) was approx. 33.46. Moreover, the OptiSecure-3D algorithm also investigated the result under noise attacks and shows better cryptanalysis results as compared to comparative state-of-art models. The findings suggest that our chaotic map-based encryption technique not only provides an effective solution to the security vulnerabilities of digital image transmission but also enhances the overall reliability of multimedia communication systems. This paper presents a significant advancement in the field of secure image encryption to meets the increasing demands for data security in modern digital communication networks.

## Introduction

With the increasing demand for internet usage and online applications, the transmission of digital documents in the form of images, video, etc. is increasing continuously. With increasing communication over insecure channels, there is also an increase in vulnerability to attacks^1^. To mitigate these concerns, researchers have developed multiple solutions such as cryptography, watermarking, etc. Therefore, secure communication is a recent evolving critical aspect of information security and privacy issues^2,3^. Image is the primary form of communication for the internet^4^. The images are majorly used in applications such as remote sensing, medical image surveillance, defense intelligence, etc^5^. Therefore, it is required to develop its security algorithm and maintain its confidentiality, integrity, and authenticity. In “Secure Image Communication”, the input image is encrypted before communicating it. This protects it from unauthorized accessing, tampering, and eavesdropping^6,7^. Several cryptographic algorithms exist such as symmetric, asymmetric, etc. that protect it from its misuse^8^. Secure key generation, encryption, and decryption are key steps of any cryptographic algorithm^9,10^. Traditional encryption methods like AES are not effective for these diverse applications that motivates the researchers to explore new techniques such as compressed sensing (CS) and chaotic systems. CS is a method that captures and recovers signals with fewer samples and is being adapted for image encryption in resource-limited environments. Various CS-based schemes enhance security through methods like hashing, permutation, and compression, aiming to protect sensitive data while managing hardware limitations. Whereas the chaotic systems provide randomness that is beneficial for cryptosystems. Techniques include dynamic DNA encoding and complex transformations to scramble and secure images. Despite the progress, existing methods still face issues such as high resource consumption and impractical key management for large-scale encryption^11–26^.

Image encryption (IE) is based on the spatial as well as frequency domains. Conventional image encryption (IE) techniques based on frequency domains such as wavelet and Fourier transforms are crucial for securing digital images. Whereas, compression and chaotic map generation depend on spatial domain. The most used method is chaos-based IE, which involves complex processes of pixel permutation and diffusion, controlled by chaotic maps. Therefore, the chaotic map has a significant role in IE. Some researchers, used metaheuristic optimizations, especially nature-inspired algorithms to enhance the efficiency of IE because it leads to complexity and long processing times^11^. In most of the recent studies, different chaotic maps, with and without optimization algorithms, have been extensively studied. While these developed schemes have shown strengths, particularly in cryptanalysis, a major limitation is the direct implementation of optimization in IE, leading to complexity and long processing times. There is dynamic relationship between cryptographic design and cryptanalysis in the development of cryptography. Cryptographic design focuses on creating new cryptographic algorithms tailored to specific problems and integrating them into practical applications^27^. Conversely, cryptanalysis examines the algorithms from an attacker’s perspective to identify security vulnerabilities. This interaction between design and analysis is crucial for enhancement of cryptographic solutions.

Therefore, there is a need for a more efficient encryption technique. Another approach such as machine learning or deep learning shows a promising and more effective solution. Leveraging its enhanced feature expression capabilities, it offers more efficient ways to encrypt images without compromising the security of the encryption systems. Therefore, motivated by this, the paper has contributions as follows:

- Paper presented a hybrid approach for secure image communication with the integration of a stacked autoencoder (SAE) and optimal 3D Arnold cat chaotic map generation termed as OptiSecure-3D.
- The integration of SAE will make the model support compressed secure image transmission.
- The optimal 3D Arnold cat chaotic map generation will provide optimal chaotic parameters that will increase the randomness of the encryption algorithm.
- Finally, the model is tested on different images taken from different resources. The result was also presented for single-image encryption as well as multiple-image encryption.

Then rest part of the paper is organized as, "Literature review" presents the literature review and comprehensive review of existing methods. "Chaotic systems" presents the preliminaries about chaotic systems. "Proposed methodology" presents the proposed methodology and discussed about each steps of the model. "Results and discussion" presents the discussion about results presented on different parameters. “Conclusion” finally presents the conclusion of the paper with future work suggestions.

## Literature review

With increased usage of internet technology, fast communication among individuals has significantly impacted various aspects of daily life. However, this raises security concerns as it harms an individual’s authenticity and potentially harms personal identity due to information leakage. To mitigate these security risks, researchers worldwide are focusing on securing communication using encryption algorithms^28–41^. Nowadays, it has been found that image data is a major source of information and may contain sensitive data that require more robust and effective security measures. Image encryption technology aims to protect images from unauthorized access. Traditional encryption methods, though effective, face challenges with efficiency, especially for large, redundant image data. Advancements in science and technology have led to more reliable and effective encryption algorithms. Among these techniques, chaotic encryption has gained attention among researchers for designing a secure image communication model due to its efficiency sensitivity, and unpredictability.

Akhtarkavan et al.^12^ introduced a novel fragile data-hiding algorithm using Integer-to-Integer Discrete Wavelet Transforms (IIDWT) and A5 Lattice Vector Quantization (LVQ) for embedding medical image Metadata and MAC, enhancing tamper detection and data embedding capacity. Bin et al.^21^ proposed a hyperchaotic image encryption model for secure communication. The model generates the secure key and performs vector operations that significantly reduce the iteration of the model and enhance the randomness. This makes the model fast and attack-resistant with lower computational complexities. Xiaoliang et al.^23^ also worked on chaotic modeling. The author presented a 3D chaotic map model for color image encryption that shuffles the pixel values and their positions. This offers a large key space and also ensures resistance to security attacks. Zhu et al.^34^ presented an artificial fish swarm algorithm with DNA coding to generate the chaotic map for image encryption. This approach addressed small key space issues and was also able to handle differential attacks.

Guan et al.^28^ developed a computational ghost imaging encryption and watermarking method with minimal key storage requirements. This method rearranges the measurement matrix using the Arnold transform and employs independent component analysis to make CGI signals statistically independent, enhancing security against cracking. The author also presented watermarking strategies for handling data tampering. Wen et al.^24^ presented a compression-based color image encryption scheme with the hybridization of chaos maps with block permutation. However, the model used the YCbCr color space. The compression is performed using discrete cosine transformation (DCT). Then on the compressed data chaotic operations are performed with the integration of block scrambling, rotation, and interchange of color components. The model presents itself as attack-resistant and shows lower computational complexity with improved efficiency. Chen et al.^37^ presented an adaptive image encryption method that utilizes the fractional order Lü system and Latin squares to enhance security against known plain image attacks (KPA) and chosen plain image attacks (CPA). This method dynamically selects Latin squares and chaotic sequences based on the sum of each bit plane and the pixel value sum of the plain image. Boussif et al.^19^ developed a ciphering algorithm using a 3D S-box for efficient image transmission, providing a significant security level and fast processing time. He et al.^16^ combined quantized synchronization of chaotic neural networks with cryptographic principles for secure image communication.

Wen and Lin^27^ identified significant security flaws in the Quantum Chaotic Map and DNA Coding Image Encryption Algorithm (QCMDC-IEA). Despite its use of chaos and DNA encoding, the algorithm’s encryption sequences are not image-dependent, making it vulnerable to cryptographic attacks due to equivalent keys and a lack of confusion and diffusion in the DNA domain. The author proposed an attack method exploiting these drawbacks, which effectively decrypts images using differential cryptanalysis and a chosen-plaintext attack, and its average breaking time is approx. 6s. Feng et al.^38^ introduced a new multi-channel image encryption algorithm called MIEA-PRHM that is based on pixel reorganization and hyperchaotic maps. This algorithm makes use of two hyperchaotic maps to generate chaotic sequences this will ultimately increases the key space and improves randomness. The encryption process involves converting input images into two fused matrices through pixel reorganization then by two rounds of scrambling and diffusion with one round of substitution on the high 4-bit matrix. The low 4-bit matrix undergoes one round of substitution and diffusion. Feng et al.^39^ introduced a hyperchaotic map named 2D-SQPM and a new efficient IE algorithm based on this map and a pixel fusion strategy (IEASP). The effectiveness of 2D-SQPM was confirmed using several chaotic indicators that shows high values of Lyapunov exponent and entropy. The IEASP algorithm enhances encryption efficiency by using common keystream that reduces the need for frequent key changes, pixel fusion to lower computational demands, and two rounds of vector-level image filtering combined with chaotic pixel superposition and quick intra-vector scrambling. Feng et al.^40^ proposed a fractional-order 3D Lorenz chaotic system coupled with a 2D sinusoidally constrained polynomial hyper-chaotic map (2D-SCPM). The fractional-order 3D Lorenz system expands the key space significantly. The MIEA-FCSM algorithm uses multi-channel fusion to reduce image pixel volume before applying two rounds of chaotic random substitution, dynamic diffusion, and fast scrambling.

Lin et al.^13^ proposed a blockchain-integrated framework for AI-generated content communication in virtual networks, enhancing security and reducing computation overhead. Xu et al.^14^ utilized the multiparty Brakerski-Fan-Vercauteren cryptosystem (D2-MHE) for secure decentralized learning systems, significantly reducing communication complexity. Fang et al.^15^ applied Takagi–Sugeno fuzzy delayed neural networks to image encryption, enhancing practicality and stability. Lv et al.^17^ focused on deep learning in service computing systems for network security, improving intrusion detection rates. Choi et al.^18^ employed compressed sensing and scrambling mechanisms for reliable image encryption, achieving strong statistical security. Sarvepalli et al.^20^ used Systematic-LT Codes over AWGN Channels for transmitting encrypted images, achieving low Bit Error Rates, and conducting thorough image quality analysis. Xinlei et al.^29^ presented a multiple-image encryption model on 2D and 3D color models. The proposed model used the 4D-chaotic system with the application of a memristor. Discrete Wavelet Transform (DWT) was used as a pre-processing step and then DNA coding was used for pixel shuffling. This model generated a more sensitive sparse measurement matrix which allows encryption as well as compression of multiple images together that use the storage space optimally. Kaur et al.^30^ developed an image encryption method using memetic differential evolution to generate keys for an intertwining logistic map. This method showed higher efficiency and security in encrypting color images compared to existing methods. Toktas et al.^32^ presented an optimal chaotic map (OCM) based image encryption technique that generates optimal chaotic parameters with a multi-objective optimization function. As compared to other approaches, this algorithm is faster and can handle cryptanalysis attacks. Wang et al.^33^ presented an image encryption approach using particle swarm optimization (PSO) to generate chaotic maps.

Kaur et al.^22^ presented compressive sensing using the hyperchaotic secure model for medical IoT networks. Additionally, the model checks the integrity by applying SHA-512. Yuan et al.^25^ presented an image encryption model for JPEG images. First AC groups are formed followed by permutation. The block permutation is the main concept of this work. The result shows reduced encryption runtime and improved generality. Lai et al.^26^ proposed a hyperchaotic map for secure image encryption. This work used the concept of fission diffusion and permutation operations. Kaur et al.^31^ presented a 4-D hyperchaotic map-based approach for secure image communication with Pareto evolutionary algorithm II to evaluate the chaotic parameter. Ma et al.^35^ combined the Tabu Search (TS) algorithm with Chen’s hyperchaos system to enhance security. The key innovation involves linking the encryption key to the ordinary image and using its hash value to initiate the hyperchaotic system that is capable to resist known-plaintext attacks. The TS algorithm is applied to scramble sub-block images optimally that will reduce the correlation among neighboring pixels and therefore enhancing the scrambling effect. Deng et al.^36^ presented a neural model that integrates a memristor with a piecewise nonlinear state function into a tabu learning neuron model. By adjusting the memristor’s state function, the model allows for easy manipulation of the chaotic butterfly’s wings. The model’s viability is further confirmed through FPGA hardware validation. Below in Table 1, the critical key feature comparison is presented to identify current approaches and their respective key strengths and limitations.

**Table 1:** Critical analysis of recent contributions to secure image communication.

Refs.	Technique	Key feature	Encryption	Watermarking	Multiple image support
^21^	Hyperchaotic map with vector operation	Hyperchaotic map	Yes	No	No
^22^	Nonsubsampled contourlet transform	Hyperchaotic map	Yes	No	No
^23^	Color image encryption algorithm	3D-chaotic map	Yes	No	No
^24^	Compression-encryption	Chaos and block permutation	Yes	No	No
^25^	Entropy encoding	Grouping coefficients	Yes	No	No
^26^	Fission diffusion process	Hyperchaotic map	Yes	No	No
^27^	Quantum chaotic map and DNA coding	DNA Coding	Yes	No	No
^28^	Ghost imaging encryption	Arnold transforms and ICA	Yes	Yes	Yes
^29^	Memristor chaotic system	DNA coding and chaotic	Yes	No	Yes
^30^	Optimal chaotic map	Memetic differential evolutionary	Yes	No	No
^31^	Optimal chaotic map	Pareto evolutionary algorithm-II	Yes	No	No
^32^	Optimal chaotic map	ABC algorithm	Yes	No	No
^33^	Chaotic	PSO	Yes	No	No
^34^	Chaotic	Artificial fish swarms	Yes	No	No

## Chaotic systems

**Fig. 1:**
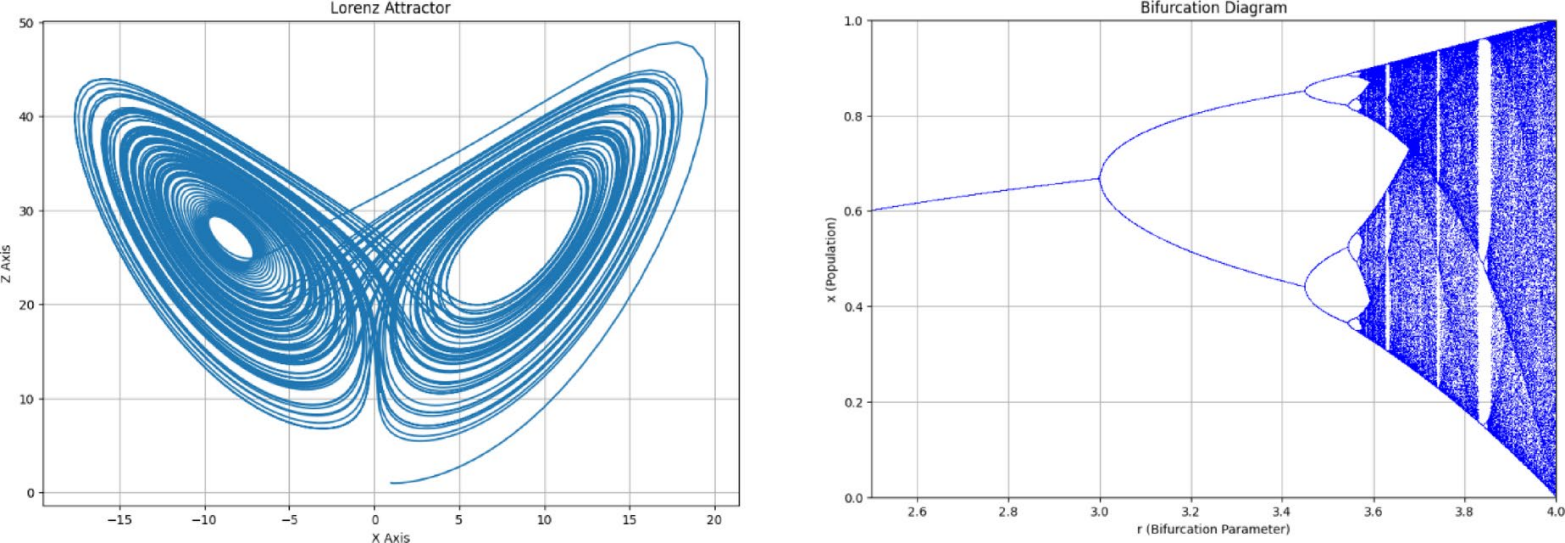
Example of (**a**) characteristics and its (**b**) bifurcation diagram of chaotic maps.

In image encryption, the chaotic systems inherent unpredictability with high internal randomness. These properties ensure that even minor changes in the encryption parameters result in massively different outcomes. Common encryption steps in chaos-based image encryption include permutation and diffusion. Scrambling is used to reduce the correlation between adjacent pixels, thereby achieving a confusing encryption effect related to pixel positions, while diffusion ensures plaintext sensitivity by spreading one or more pixels’ values to other pixels, thereby helping to resist differential attacks^41,42^. The “chaos” term was introduced by Lorenz in the 1900s for making decisions in unpredictable complex environments. This motivated the researchers to use the chaotic behavior in the encryption process to handle sensitive information. In 1997, Fridrich^43^presented an image encryption algorithm using chaotic maps for secure data transmission. As stated by the author, Chaos theory is extremely sensitive to initial conditions and parameters. This means that a small change in data may lead to significantly different outcomes. There are several algorithms to generate chaos. One of them is Hénon Map^44^. It is a type of discrete-time dynamic system that operates by mapping a point $\:\left({x}_{i},{y}_{i}\right)$ to a new point $\:\left({x}_{i+1},{y}_{i+1}\right)$. Mathematically it is represented as:

1 $$\:{x}_{i+1}=1-a{x}_{i}^{2}+{y}_{i},$$

2 $$\:{y}_{i+1}=b{x}_{i},$$

where $\:a\:and\:b$are the parameters to generate chaotic maps. Another algorithm for the generation of chaotic maps is the Lorenz chaotic map^45^. This was developed by Edward Lorenz and is formed by a set of differential equations characterized by attractors. This gives the arrays of chaotic solutions^46^. In Fig. 1, an example of the characteristics diagram and bifurcation diagram is presented for Lorenz chaotic maps. Mathematically, Lorenz chaotic maps are generated as:

3 $$\begin{aligned}\frac{dx}{dy}=a(y-x) \\\:\frac{dy}{dt}=rx-y-xz\\ \frac{dz}{dt}=xy-b. \end{aligned}$$

Here, the value of $\:x,\:y,\:and\:z$ plays a crucial role in the initial steps as they generate the specific chaotic maps and are also used in permutation. Whereas $\:a,\:r\:and\:b$are the chaotic parameters. Alternatively, the Arnold Map is also used for the generation of 2D chaotic maps. It is based on the following transformation^47,48^:

4 $$\:\left(\begin{array}{c}{x}_{i+1}\\\:{y}_{i+1}\end{array}\right)=\left(\begin{array}{cc}1&\:a\\\:b&\:ab+1\end{array}\right)\left(\begin{array}{c}{x}_{i}\\\:{y}_{i}\end{array}\right)mod\:1,$$

where $\:a\:and\:b$ are chaotic parameters. This modeling can be extended for 3D image encryption by introducing a third variable $\:{z}_{i}$​. This adaptability of Arnold modeling makes it versatile for secure image communication.

## Proposed methodology

**Fig. 2:**
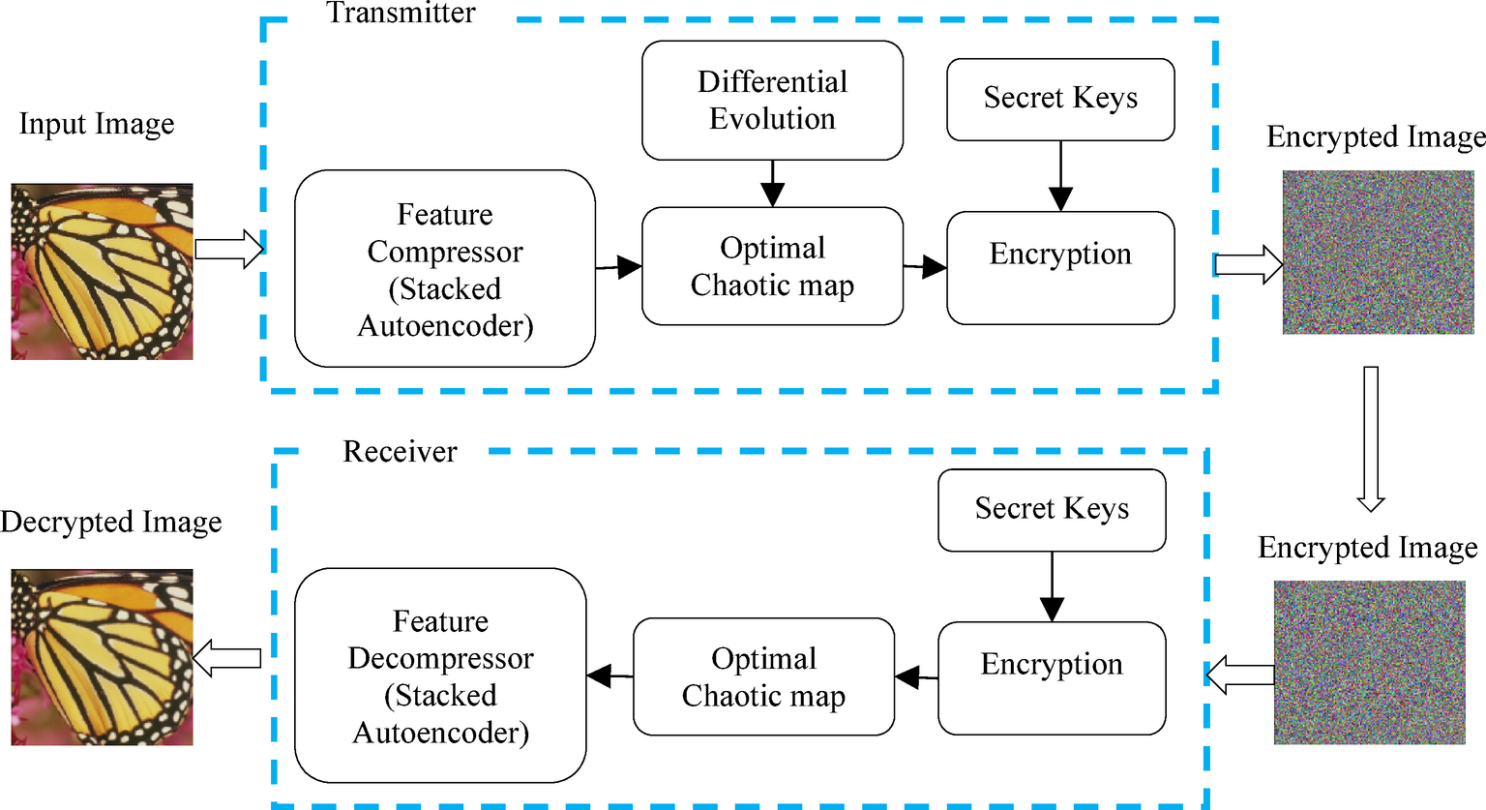
Proposed methodology.

In this section, an image encryption methodology is proposed OptiSecure-3D for secure communication. The methodology is designed using an optimized parameter-based 3D chaotic map for image encryption. The OptiSecure-3D model is presented in Fig. 2. The OptiSecure-3D image encryption method integrates three primary components: SAE, optimized parameter-based chaotic mapping, and encryption/decryption module, to ensure robust and secure encryption of images. These components are described below in sub-sections. The algorithm is presented below in Algorithm 1.

**Algorithm 1:**
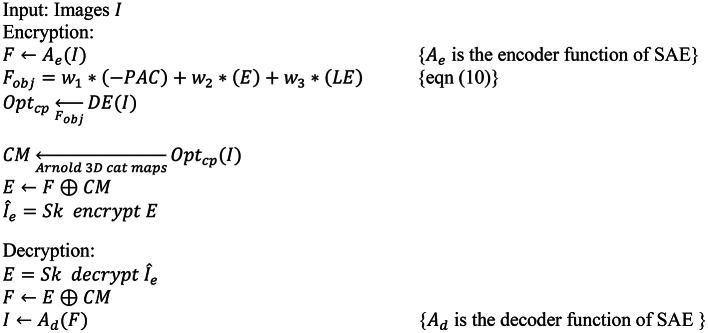
Proposed methodology.

### High dimensional feature compression and decompression using SAE

Initially, the SAE is used to extract complex and high-dimensional features $\:HDF$ in images. Autoencoders are neural network structures with three primary layers: an input layer, a hidden layer, and an output layer. The training process of an autoencoder is divided into two parts: encoding and decoding. The encoder (compressed) maps input data to hidden representations. This process is described by the formula $\:{h}_{n}=f({X}_{1}{l}_{n}+{d}_{1})$, where $\:f$ is the encoding function, $\:{X}_{1}$​ is the encoder weight matrix, and $\:{d}_{1}$​ is the bias vector. The decoder (decompressor) reconstructs input data from these encrypted (or encoded) representations, following the formula $\:{A}_{n}=g({X}_{2}{l}_{n}+{d}_{2})$, where $\:g$ is the decoding function, $\:{X}_{2}$​ is the decoder weight matrix, and $\:{d}_{2}$​ is another bias vector. The parameters of the autoencoder are updated to minimize the reconstruction error.

### Optimized parameters based 3D chaotic map

**Fig. 3:**
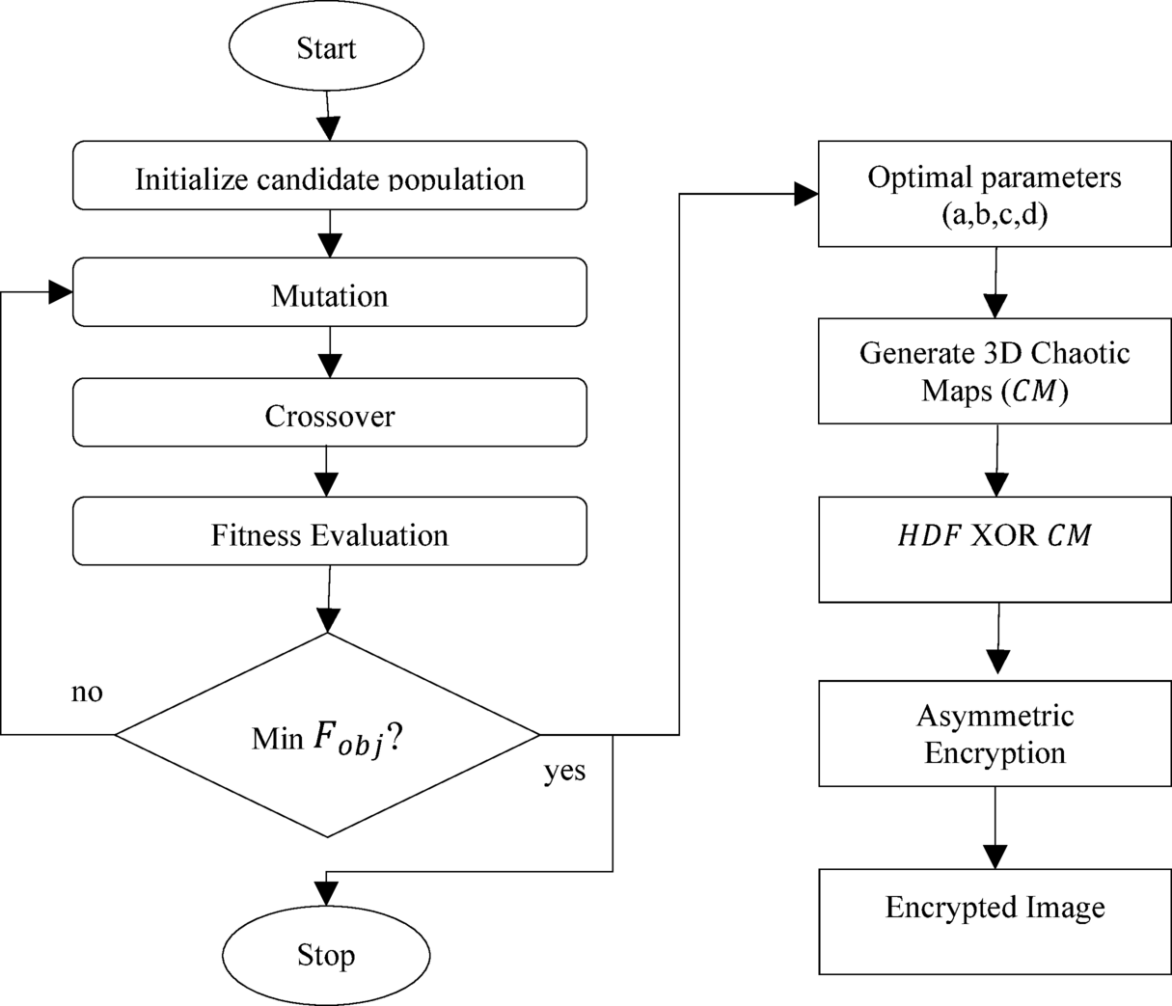
Proposed optimized parameters based on 3D Chaotic Map.

In this step, the optimized parameter-based chaotic map is generated. The OptiSecure-3D chaotic map generation flowchart is presented in Fig. 3. The base of the proposed optimized chaotic map generation is Arnold’s cat map. Arnold’s cat map is named after Vladimir Arnold, who illustrated its effects using a cat image in the 1960s^49^. It randomly permutates the image to encrypt it. However, repeating the map a specific number of times will revert the image to its original form. The 2D representation of the chaotic map is mathematically represented as:

5 $$\:\left(\begin{array}{c}\widehat{x}\\\:\widehat{y}\end{array}\right)=\left(\begin{array}{cc}1&\:a\\\:b&\:ab+1\end{array}\right)\left(\begin{array}{c}x\\\:y\end{array}\right)mod\:N,$$

where $\:a\:and\:b$ represents the chaotic parameters. $\:N$ represents the size of the input image. The 3D Arnold cat map is an extension of the 2D version. It is achieved by adding 2 more parameters such as $\:c\:and\:d$ to make it more complex transformations in three-dimensional space presented as:

6 $$\:\left(\begin{array}{c}\begin{array}{c}\widehat{x}\\\:\widehat{y}\end{array}\\\:\widehat{z}\end{array}\right)=\left(\begin{array}{ccc}1&\:0&\:a\\\:bc&\:1&\:abc+c\\\:bcd+b&\:d&\:abcd+ab+cd+1\end{array}\right)\left(\begin{array}{c}\begin{array}{c}x\\\:y\end{array}\\\:z\end{array}\right)mod\:N,$$

When the inverse transform is applied in Eq. (6) then the original matrix is obtained as:

7 $$\:\left(\begin{array}{c}\begin{array}{c}x\\\:y\end{array}\\\:z\end{array}\right)=\left(\begin{array}{ccc}1+ab&\:ad&\:-a\\\:0&\:1+cd&\:-c\\\:-b&\:-d&\:1\end{array}\right)\left(\begin{array}{c}\begin{array}{c}\widehat{x}\\\:\widehat{y}\end{array}\\\:\widehat{z}\end{array}\right)mod\:N.$$

The parameters used in Eq. (6) are obtained using a differential evolution optimization algorithm.

The differential evolutionary algorithm is inspired by the natural evolution of living organisms. It is a method used for optimizing a problem with key steps such as mutation, crossover, and selection. It consists of the following steps:

- Initial Phase: In this step, chromosomes are initialized. In the initial phase, the chromosome population is initialized randomly to generate a candidate population which is specified under the lower and upper bounds as $\:{L}_{b}{x}_{ij}\:and\:{U}_{b}{x}_{ij}$, respectively. This is termed as the initial population.
- Mutation Phase: In this phase, a new variable is created using mutation with mutation factor $\:F$. It is evaluated as: $\:{x}_{i+1}={x}_{i}+F$.
- Crossover Phase: In this phase, the mutated variables are exchanged randomly with previous generations with a cross-over rate $\:C$. This will result in a new variable for a new generation.

These phases are repeated sequentially until a pre-defined maximum number of generations is reached. The goal of the DE algorithm is to find the optimal solution to a problem by simulating the process of natural selection, where only the fittest solutions survive and evolve. Here, the fitness function is designed using multi-objective functions. This objective function is designed based on adjacency correlation, entropy, and the Lyapunov exponent.

Pixel Adjacency Correlation ($\:PAC$): It is a measure that obtains correlation among pixels of shuffled images by averaging over all pairs of pixels vertically, horizontally, and diagonally^50^. A lower correlation shows better shuffling. Therefore, the minimization function is considered for PAC. Mathematically, it is evaluated as :

8 $$\begin{aligned}PAC= & \frac{1}{M(N-1)}\\ & \sum_{i=1}^{M}\sum_{j=1}^{N-1}\frac{({x}_{ij}-{x}_{\mu\:})({x}_{ij+1}-{x}_{\mu\:})}{{\sigma\:}^{2}},\end{aligned}$$

where $\:M\:and\:N$ is the size of the input image $\:I$. $\:{x}_{ij}$ represents the total pixels with variance $\:{\sigma\:}^{2}$ and $\:{x}_{\mu\:}$ is the mean pixel value.

Entropy ($\:E$): It is the measure to evaluate the randomness of shuffled images^51^. The higher the entropy, the more secure the encryption. Therefore, the minimization function is considered for PAC. Mathematically, it is evaluated as:

9 $$\:E=-{\sum\:}_{x}{p}_{x}{log}_{2}{p}_{x},$$

where, $\:{p}_{x}$ represents the probability of the intensity level $\:x$.

Lyapunov Exponent ($\:LE$): It is the measure to evaluate the sensitivity of the system to initial conditions. A higher Lyapunov exponent indicates a more chaotic system^52^.

Multi-Objective Function ($\:{F}_{obj}$): By combining all these functions into a single objective function with a weight function $\:{w}_{1},\:{w}_{2},\:and\:{w}_{3}$ respectively.

10 $$\:{F}_{obj}={w}_{1}*\left(-PAC\right)+{w}_{2}*\left(E\right)+{w}_{3}*\left(LE\right).$$

Here, $\:-PAC$ is used because the goal is to minimize correlation, while E and LE are maximized.

This function can then be used as the fitness function in the evolutionary approach to find the optimal parameters for the Arnold 3D Cat Maps.

To explain it we take here a small example. This example explains the Differential Evolution (DE) algorithm for optimizing the parameters and for Arnold’s Cat Map. Let N be 256. Using Eq. (5), the goal is to optimize the parameters and to maximize entropy. Then an initial population of candidate solutions with a and b are randomly chosen between 1 and *N* − 1 such as: Candidate 1: ($\:{a}_{1}$, $\:{b}_{1}$)=(50,100), Candidate 2: ($\:{a}_{2}$, $\:{b}_{2}$)=(150,200), Candidate 3: ($\:{a}_{3}$, $\:{b}_{3}$)=(75,125) and Candidate 4: ($\:{a}_{4}$, $\:{b}_{4}$)=(25,225). For each candidate, mutation is performed such as

$$\begin{aligned} & (a_m, b_m) = ( a_2, b_2)+0.8 \times ((a_3, b_3) - ( a_4, b_4))\\ & (a_m, b_m)=(150,200)+0.8 \times ((75-25,125-225)) \\ & ({a}_{m},\:{b}_{m})=\left(\text{150,200}\right)+(40, - 80)\\&(a_m, b_m) = (190,120) \end{aligned}.$$

For Candidate 1, we mix (a1,b1) and ($\:{a}_{m}$, $\:{b}_{m}$) using the crossover rate. Suppose for $\:a$ with probability 0.7, we choose $\:{a}_{m}$​, otherwise $\:{a}_{1}$​. For $\:b$ lets suppose $\:{b}_{1}$ is chosen​. Then the vector will be $\:{a}_{t},{b}_{t}=\left(\text{190,100}\right).$

Suppose the chaotic behavior score for (190,100) is higher than for (50,100). Then, we replace Candidate 1 with the new trial vector. Then, same process is repeated with other candidates. The entire process is repeated for the specified number of generations to obtain best chaotic behavior score.

### Encryption

In this step, the encryption process is performed. It is performed after optimal chaotic map generation. The compressed features extracted $\:Comp\left({I}_{p}\left(x,y,z\right)\right)\:$out of the SAE are xored with the extracted optimal chaotic maps $\:{C}_{m}\left(x,y,z\right)$as:

11 $$\:{I}_{e}\left(x,y,z\right)=Comp\left({I}_{p}\left(x,y,z\right)\right)\oplus\:\:{C}_{m}(x,y,z).$$

This xor operation will result in encrypted chaotic keys $\:{I}_{e}\left(x,y,z\right)$. This operation will add randomness and non-linearity properties and ultimately increase the complexity of the encryption process.

Following this, asymmetric encryption is performed for secure communication over the generated chaotic keys $\:{I}_{e}\left(x,y,z\right)$ generated in Eq. (11).

12 $$\:{\widehat{I}}_{e}\left(x,y,z\right)=Sk\:\:encrypt\:{I}_{e}\left(x,y,z\right),$$

where, secure keys $\:Sk$ are used to perform encryption. While for decryption the inverse process is performed for Eqs. (12) and (11).

## Results and discussion

The performance assessment of the proposed OptiSecure-3D is covered in this section. The experimental investigation was performed on an Intel Core i5 processor with an 8 GB hard drive. Using the Python platform, simulation analysis was carried out. For this experiment analysis, tensorflow and matplotlib were used with cryptography library. To evaluate the performance of the OptiSecure-3D work following tests are evaluated as presented in sub-sections. The model is tested on a benchmark dataset set5^61^, set14^61^, and MRI^62^ and apart from this, other images are also taken from different areas such as medical images.

### Information entropy evaluation

**Fig. 4:**
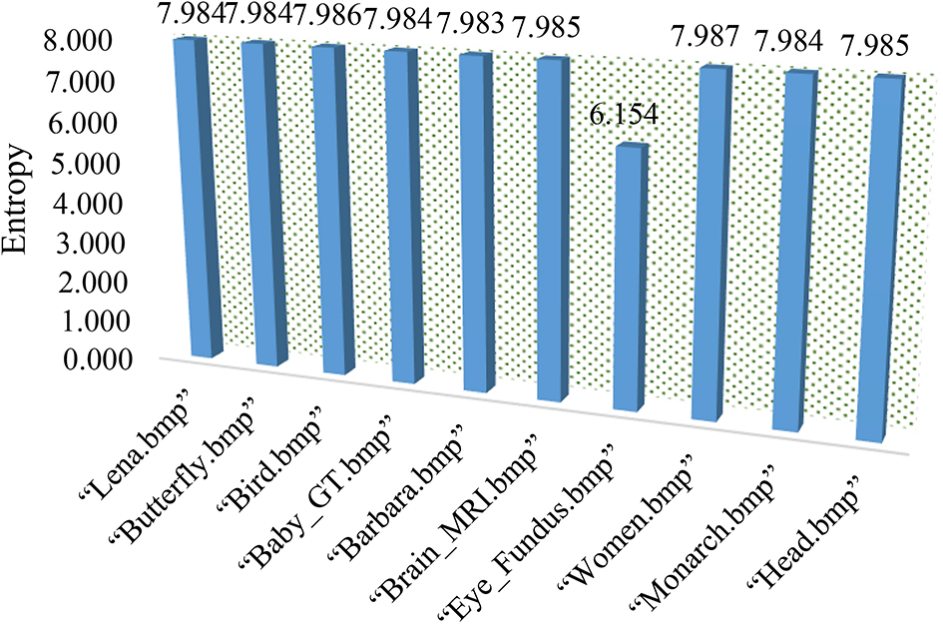
Entropy evaluation.

**Fig. 5:**
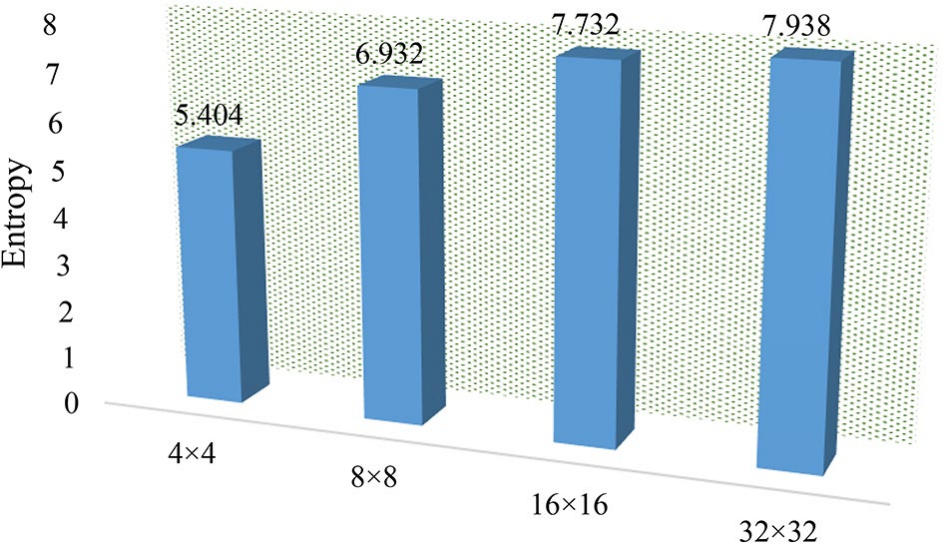
Local entropy evaluation.

Information entropy (IE) is a key metric for the evaluation of the randomness of an image, and it’s particularly crucial in the context of image encryption^38^. The closer the entropy value is to 8, the more randomized or diffused the ciphertext image is, indicating a higher level of security. The mathematical representation of entropy is represented in Eq. (9). Below in Fig. 4, some images are tested on a OptiSecure-3D algorithm and its entropy outcome is represented graphically. All encrypted image values are quite close to theoretical value. Such high entropy levels are indicative of strong encryption, making the images more secure against cyberattacks. Below in Fig. 5 the local entropy assessment is presented for image encryption based on different image sub-block sizes, with four categories such as 4 × 4, 8 × 8, 16 × 16, and 32 × 32. From the graph, it is observed that as the sub-block size increases, the entropy also increases that will provide a stronger encryption due to increased data randomness. This can be important for applications requiring robust security measures to protect image data from unauthorized access or analysis.

### Peak signal-to-noise ratio

PSNR (Peak Signal-to-Noise Ratio): In this work, PSNR is used to evaluate the quality of the encrypted image as compared to the original image^38^. Therefore, its lower value represents its efficacy.

13 $$\:PSNR=10.{log}_{10}\left(\frac{{Max}_{i}^{2}}{MSE}\right).$$

MSE (Mean Squared Error): Measures the average of the squares of the errors between the estimated and actual values. Lower MSE values generally indicate better image quality.

14 $$MSE=\frac{1}{N}\sum_{i=1}^{N}{({O}_{i}-{P}_{i})}^{2},$$

where N is the total number of pixels, $\:{O}_{i}$ is the original image and $\:{P}_{i}$ is the decrypted image.

**Fig. 6:**
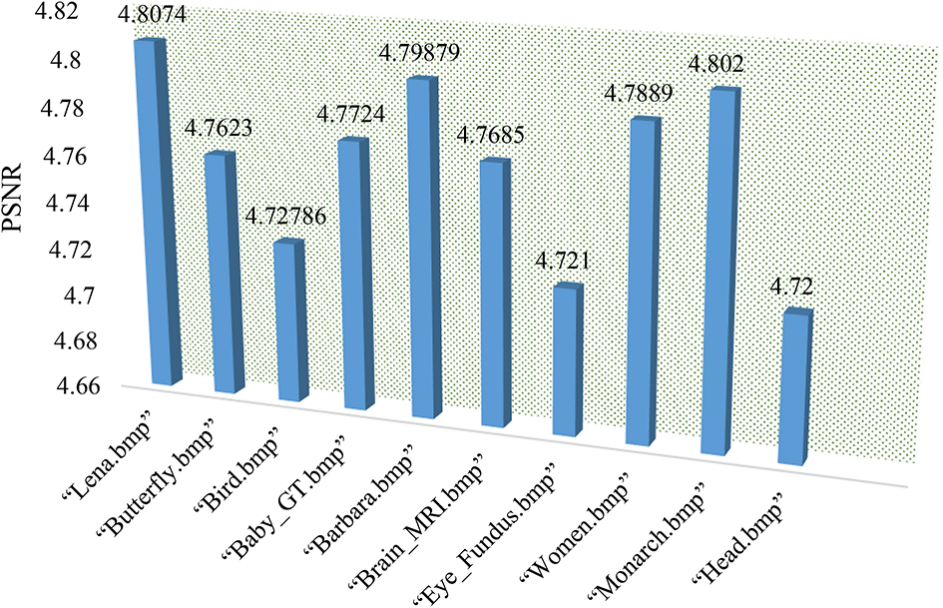
PSNR evaluation.

Below in Fig. 6, the PSNR evaluation of some example images is presented.

### Randomness tests

**Fig. 7:**
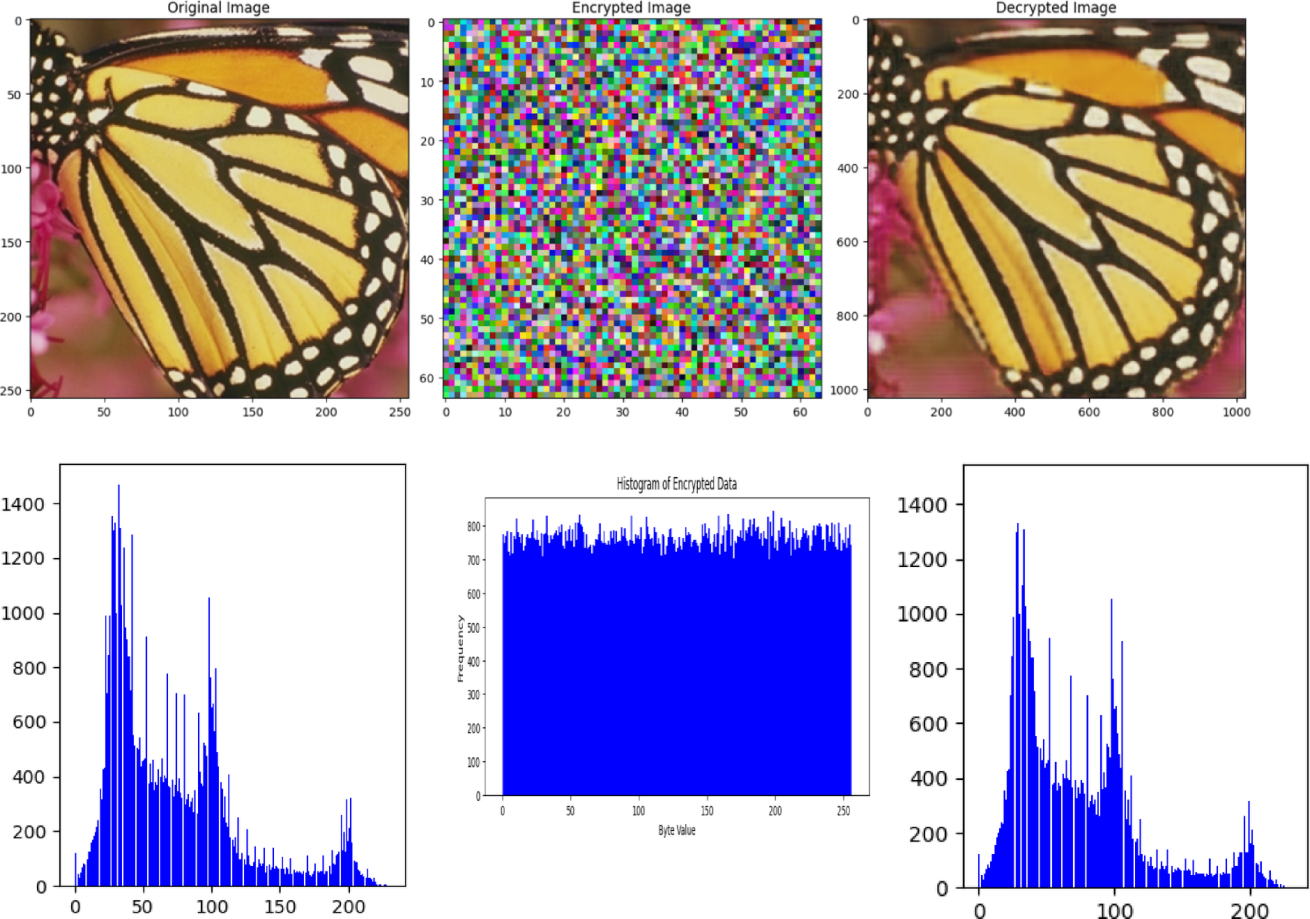
Plain (left), encrypted (center), decrypted (right) histogram analysis.

One of the requirements of the encryption algorithm is its randomness test. This section provides a visual examination and statistical test for proof of proposed OptiSecure-3D encryption robustness. The visual analysis of the encrypted images and their histograms, presented in Fig. 7 indicates effective randomization. Figure 7 illustrates the histograms of pixel values for plain, encrypted, and decrypted images. It demonstrates that while the plain image’s pixel values display a discernible pattern, the encrypted image’s pixel values are distributed in a manner that closely approximates a uniform distribution. This contrast highlights the effectiveness of the encryption process in disguising the original patterns in the data, a key aspect of strong encryption. Below in Table 2, the statistical test results are presented for five sample images. The entropy values are consistently high across all images, indicative of excellent randomness. The pairwise-byte frequency test also shows high values for most images, further suggesting good randomness. The statistical tests, particularly the high entropy and favorable chi-squared results, support this by demonstrating a high level of randomness. Although the pairwise byte frequency tests indicate good randomization, they suggest the need for further detailed analysis. Therefore, these results collectively point towards the effectiveness of the proposed OptiSecure-3D algorithm in achieving robust and secure encryption.

**Table 2:** Statistical test result for randomness test.

Tests/Images	“Lena.bmp”	“Butterfly.bmp”	“Bird.bmp”	“Baby_GT.bmp”	“Barbara.bmp”
Frequency	48	48	48	48	48
Chi-squared	270.25	258	245.7	269.2	294.3333
Entropy	7.984	7.9847	7.985	7.984	7.982
Pairwise-byte frequency	11,179	11,191	11,214	11,180	11,163
p-value	0.2445	0.4357	0.6505	0.258	0.0456

### Pixel adjacency correlation test

**Fig. 8:**
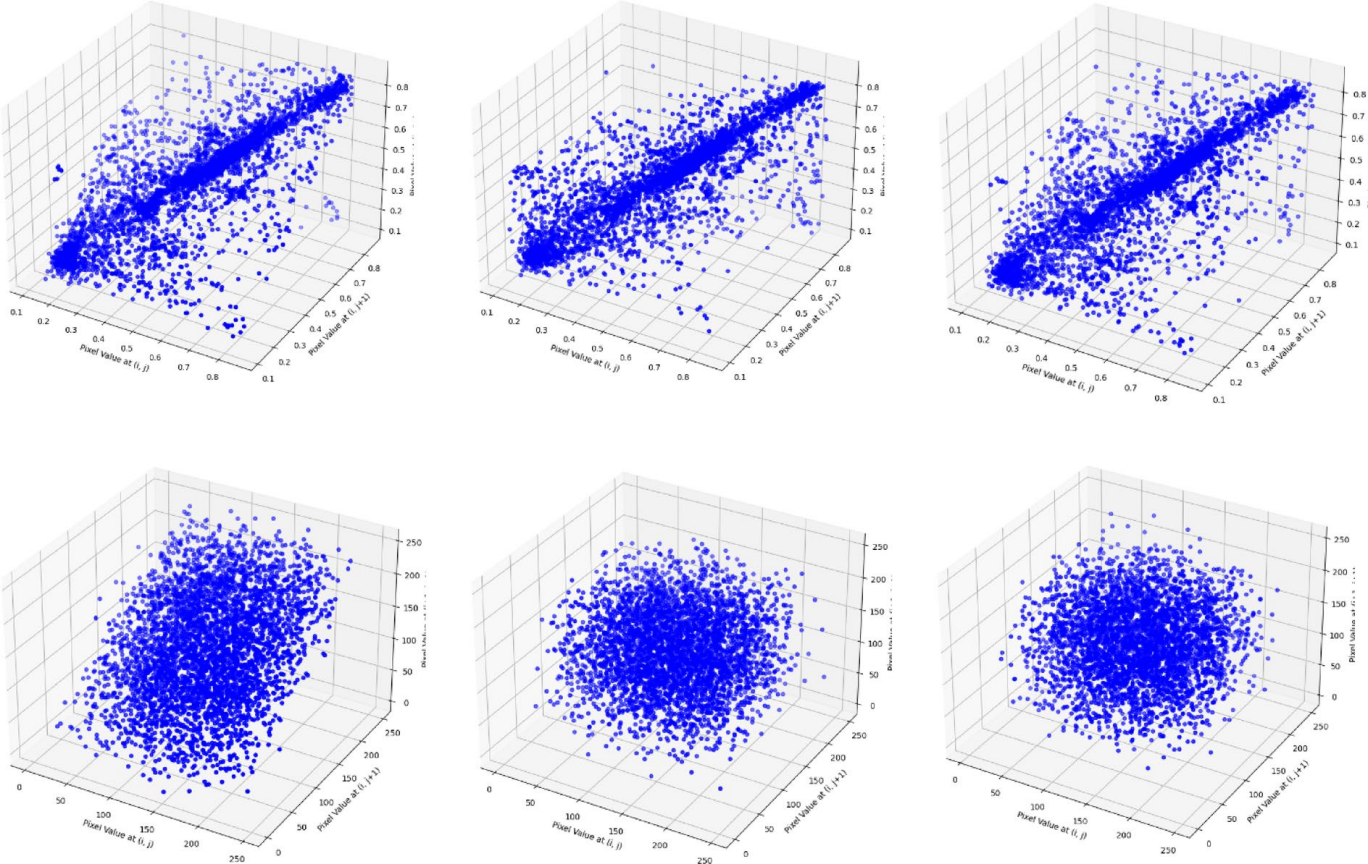
Adjacent pixel correlation of (**a**) original image (top), (**b**) encrypted image (bottom).

In regular images, the adjacent pixels are quite correlated horizontally, vertically, and diagonally. In ordinary images, the correlation is near about 1. However during secure communication using the image encryption technique, the pixel adjacency correlation test (PACT) is evaluated because a good encryption algorithm shows very little correlation among adjacent pixels and resists statistical attack if the correlation coefficients are very low^63^. Therefore, it is used to evaluate the security strength among pixels. Mathematically, it is evaluated as:

15 $$\:PACT=\frac{\sum\:_{i=1}^{n}({X}_{i}-\widehat{X})({Y}_{i}-\widehat{Y})}{\sqrt[2]{\sum\:_{i=1}^{n}{({X}_{i}-\widehat{X})}^{2}\sum\:_{i=1}^{n}{({Y}_{i}-\widehat{Y})}^{2}}},$$

where, $\:{X}_{i}$ represents the pixel value in the original image and $\:{Y}_{i}$ represents the pixel value in the encrypted image with $\:\widehat{X}$ and $\:\widehat{Y}$ mean value respectively. An example of a correlation plot is presented below in Fig. 8. It is evaluated among plaintext and ciphertext. The Fig. 8(a) represents the adjacent pixel correlation is presented for the original image whereas Fig. 8(b) presents the pixel correlation for encrypted images. Table 3 presents some sample image outcomes for correlation among horizontal, vertical, and diagonal pixels.

**Table 3:** Pixel adjacency correlation test.

Tests/Images	Horizontal	Vertical	Diagonal
“Lena.bmp”	−0.00878	−0.0111	−0.00868
“Butterfly.bmp”	−0.0035	0.0023604	−0.013
“Bird.bmp”	0.00085	−0.00369	−0.01026
“Baby_GT.bmp”	−0.00603	−0.0105	0.0073
“Barbara.bmp”	−0.00369	−0.00331	−0.00362
“Brain_MRI.bmp”	−0.0113	−0.00052	−0.00012
“Eye_Fundus.bmp”	0.0138	−0.024	−0.024
“Women.bmp”	0.0095	−0.006	0.0001
“Monarch.bmp”	−0.00184	−0.0006	0.0016
“Head.bmp”	0.005	0.0083	−0.0134

### Differential analysis test

Differential Analysis (DA) test shows the importance of encryption robustness against differential or chosen-plaintext attacks, where an attacker modifies a single pixel in the plain image and examines the changes in the encrypted version to discover vulnerabilities. A strong encryption algorithm should ensure that even the smallest change in the original image, like altering a single bit, results in significant and unpredictable alterations in the encrypted image. Two major parameters for differential testing are the Number of Pixels Change Rate (NPCR) and Unified Average Changing Intensity (UACI) are the metrics to test differential analysis attacks.

NPCR: This measure is used to check the percentage of changed pixels in an encrypted image when a single pixel is changed in the original image. A high value of NPCR represents a high sensitivity change in image, which is desired in secure encryption algorithms^38^. Mathematically it is evaluated as:

16 $$\:NPCR=\frac{\text{N}\text{u}\text{m}\text{b}\text{e}\text{r}\:\text{o}\text{f}\:\text{D}\text{i}\text{f}\text{f}\text{e}\text{r}\text{e}\text{n}\text{t}\:\text{P}\text{i}\text{x}\text{e}\text{l}\text{s}}{\text{T}\text{o}\text{t}\text{a}\text{l}\:\text{N}\text{u}\text{m}\text{b}\text{e}\text{r}\:\text{o}\text{f}\:\text{P}\text{i}\text{x}\text{e}\text{l}\text{s}}*100.$$

UACI: It is a measure to evaluate the average difference in the intensity between the original and encrypted images. An ideal UACI value is theoretically evaluated by^53^, which is 33.4635. Mathematically, UACI is evaluated as:

17 $$\begin{aligned}UACI=&\frac{1}{n*m}\\&\sum_{i=1}^{n}\sum_{j=1}^{m}\frac{{P}_{ij}-{C}_{ij}}{L}*100,\end{aligned}$$

$\:{P}_{ij}$ is the original image and $\:{C}_{ij}$ is the encrypted image.

Below in Table 4, some example images and their respective NPCR and UACI are presented. This table shows the key sensitivity analysis of the proposed OptiSecure-3D model. Key sensitivity in an image encryption context refers how radically the encrypted output changes when the key is modified by a very small amount. In Table 4, the NPCR and UACI values for various test images consistently remain high for sample images. These metrics indicate that even the slightest alteration in the key leads to significant changes across nearly all pixels in the encrypted image. Such high NPCR and UACI values demonstrate strong key sensitivity that shows an attacker cannot rely on a key that is even marginally incorrect to retrieve any meaningful information from the ciphertext. This robust sensitivity is a crucial cryptographic property that provides strong security against attacks.

The Table 5 presents the NPCR and UACI values across different image sizes. Both NPCR and UACI values show low standard deviations that indicates uniform performance of the encryption across different images and sizes (Table 5).

**Table 4:** NPCR and UACI evaluation.

Tests/Images	NPCR	UACI
“Lena.bmp”	99.54	0.334688
“Butterfly.bmp”	99.35	0.334687
“Bird.bmp”	99.98	0.33467
“Baby_GT.bmp”	99.89	0.33469
“Barbara.bmp”	99.99	0.33467
“Brain_MRI.bmp”	99.76	0.33469
“Eye_Fundus.bmp”	99.85	0.33469
“Women.bmp”	99.89	0.3346
“Monarch.bmp”	99.91	0.3347
“Head.bmp”	99.99	0.334688

**Table 5:** NPCR and UACI evaluation with variation of different input size.

Image Size	128 × 128		256 × 256		512 × 512		1024 × 1024	
Image	NPCR	UACI	NPCR	UACI	NPCR	UACI	NPCR	UACI
“Lena.bmp”	99.54	0.33468	99.56	0.3342	99.43	0.3349	99.62	0.335
“Brain_MRI.bmp”	99.76	0.3346	99.74	0.3348	99.78	0.3344	99.64	0.3348
“Eye_Fundus.bmp”	99.85	0.3346	99.81	0.3341	99.88	0.3347	99.83	0.3345
Standard Deviation	0.1302	0.0000377	0.1053	0.00031	0.19293	0.00021	0.09463	0.00021

### Noise attack test

In this section, the result evaluation under gaussian noise attack is presented. The PSNR is evaluated for cipher image (CI) and decrypted image (DI). Below in Table 6, the result is presented with different noise levels (Standard deviation = 10, 15, 20, and 25). The PSNR of CI is evaluated between the original image and the attacked encrypted image. Whereas the PSNR of DI is evaluated between the original image and the decrypted image after the attack. From the result, an average CI was approx. 4 and the average PSNR was approx. 27. The standard deviation for evaluated PSNR is quite low that shows the proposed model is more robust. The Table 7 provides PSNR evaluation for different images under varying probability of salt and pepper noise. The PSNR values for the DI are consistently high across all images and noise levels. This indicates a robust resistance to noise. The CI values are significantly lower. The standard deviation values provided for both CI and DI across different noise intensities are low this exhibits a strong resilience to salt and pepper noise.

**Table 6:** PSNR evaluation under Gaussian noise attack.

	CI	DI	CI	DI	CI	DI	CI	DI
	10		15		20		25	
“Head.bmp”	4.775	27.905	4.7857	27.91	4.796	27.9138	4.809	27.9139
“Eye_Fundus.bmp”	4.735	27.9264	4.7425	27.915	4.783	27.8784	4.8068	27.88
“Brain_MRI.bmp”	4.79	27.881	4.785	27.86	4.812	27.916	4.825	27.935
“Lena.bmp”	4.7828	27.88	4.804	27.8762	4.75	27.91	4.774	27.897
“Butterfly.bmp”	4.7436	27.91	4.816	27.863	4.8365	27.897	4.7787	27.858
Standard Deviation	0.0219062	0.017787	0.024961	0.023287	0.028914	0.01397	0.019357	0.026606

**Table 7:** PSNR evaluation under salt and pepper attack.

	CI	DI	CI	DI	CI	DI
	0.01		0.05		0.1	
“Head.bmp”	4.76	27.84	4.81	27.86	4.82	27.88
“Eye_Fundus.bmp”	4.73	27.909	4.74	27.927	4.784	27.935
“Brain_MRI.bmp”	4.788	27.89	4.77	27.86	4.760	27.89
“Lena.bmp”	4.810	27.86	4.79	27.86	4.83	27.912
“Butterfly.bmp”	4.77	27.88	4.774	27.89	4.76	27.86
Standard Deviation	0.0268745	0.023886	0.023172	0.026485	0.029437	0.025935

### Comparative state-of-art

**Fig. 9:**
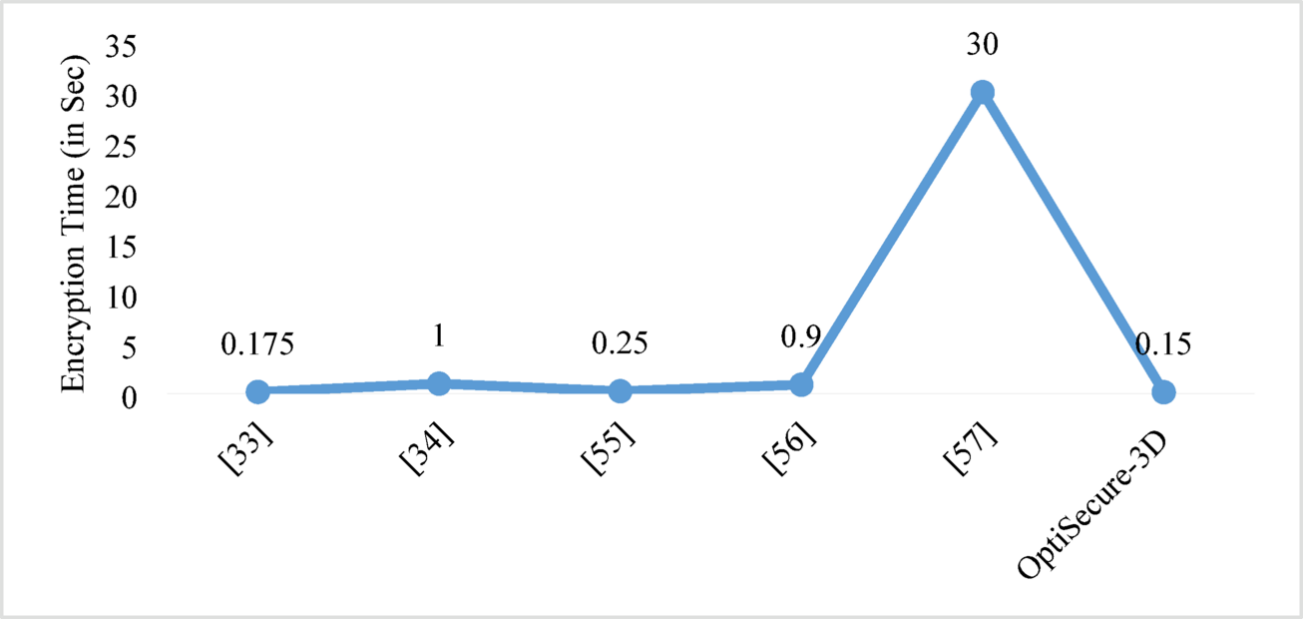
Comparative encryption time analysis.

The OptiSecure-3D approach employs a comprehensive set of widely recognized performance metrics such as information entropy, PSNR, NPCR, UACI, and pixel adjacency correlation which are standard benchmarks in image encryption research. By using these established tests, the assessment is directly comparable to existing works that will ensure the findings have been validated over time. In Table 8, a comparative state-of-art is presented for image encryption techniques. Authors in^54–60^ motivated how to perform a comprehensive comparison with recent state-of-the-art solutions to enhance the scientific soundness and persuasiveness of the OptiSecure-3D. These techniques predominantly utilize higher-dimensional encryption methods, ranging from 1D to 4D, indicating a trend toward more complex and secure encryption. All methods employ chaotic systems, reflecting their popularity due to unpredictability and sensitivity, essential for effective encryption. There’s a diversity in the optimization algorithms used, such as Memetic Differential Evolutionary^30^, Pareto Evolutionary Algorithm-II^31^, ABC algorithm^32^, PSO^33^, and Artificial fish swarms^34^ to enhance encryption quality. All techniques focus on single-image encryption. Performance-wise, these methods consistently achieve high entropy and low correlation, crucial for robust encryption, with NPCR values above 99.5% and UACI values around 33% which is near to the theoretical value^53^. This analysis underscores a continuous evolution in image encryption, marked by increasing complexity, integration of innovative optimization algorithms, and the recent inclusion of compression features, collectively enhancing the security and efficiency of image encryption solutions. With an observed NPCR of 99.89 critical value test is performed with significance level of 95%. The result shows the z-score valye greater than 1.96 that indicates that it is statistically significant. Every pixel in the encrypted image changed relative to the original when a single pixel was modified in the plaintext image. This is a strong indication that the encryption algorithm is highly sensitive to changes in the input that is a desirable property in cryptographic systems because it suggests a high level of resistance to differential attacks. This also prevent attackers from predicting how changes in the plaintext affect the ciphertext. Figure 9compares the encryption time (in sec) of various existing techniques with the OptiSecure-3D. It is evident that the OptiSecure-3D algorithm, requiring only 0.15 s that outperforms all other approaches in terms of speed. While the closest methods achieve encryption times of 0.175 s^32^and 0.25 s^48^, the OptiSecure-3D technique still shows a notable improvement. These findings underscore the efficiency gains offered by the OptiSecure-3D encryption strategy.

**Table 8:** Comparative state-of-art.

Refs.	Year	Dimension	Chaotic	Optimization	Compression	Single image	Multi image	Entropy (mean)	Corr (mean)	NPCR	UACI
^30^	2019	3D	Logistic	Memetic differential evolutionary	No	Yes	No	7.9987	0.0032	99.6535	33.5497
^31^	2020	4D	Lorentz	Pareto evolutionary algorithm-II	No	Yes	No	7.9976	0.00372	99.634	33.433
^32^	2021	1D	Chaotic	ABC algorithm	No	Yes	No	7.9940	0.00013	99.609	33.46
^33^	2022	2D	Chaotic	PSO	No	Yes	No	7.9978	0.00092	99.595	33.406
^34^	2023	2D	Chaotic	Artificial fish swarms	No	Yes	No	7.9992	0.0036	99.62	33.69
Proposed		3D	Chaotic	Differential Evolutionary	SAE	Yes	Yes	7.9984	0.005	99.89	33.468

**Table 9:** Comparative state-of-art.

Ref	Year	Dimension	Chaotic	Optimization	Compression	Single image	Multi Image	Entropy (mean)	Corr (mean)	NPCR	UACI
^30^	2019	3D	Logistic	Memetic differential evolutionary	No	Yes	No	7.9987	0.0032	99.6535	33.5497
^31^	2020	4D	Lorentz	Pareto evolutionary algorithm-II	No	Yes	No	7.9976	0.00372	99.634	33.433
^32^	2021	1D	Chaotic	ABC algorithm	No	Yes	No	7.9940	0.00013	99.609	33.46
^33^	2022	2D	Chaotic	PSO	No	Yes	No	7.9978	0.00092	99.595	33.406
^34^	2023	2D	Chaotic	Artificial fish swarms	No	Yes	No	7.9992	0.0036	99.62	33.69
OptiSecure-3D		3D	Chaotic	Differential evolutionary	SAE	Yes	Yes	7.9984	0.005	99.89	33.468

## Conclusion

The proposed 3D-optimized chaotic map-based image encryption is modeled with SAE and differential optimization to support compressed encryption with optimal chaotic parameters. The optimized chaotic parameters are evaluated on a multi-objective function. The OptiSecure-3D IE resists numerous cryptanalyses and quantitative results were validated against state-of-the-art models. The result evaluated the OptiSecure-3D secure image encryption algorithm with a randomness test, pixel adjacency correlation test, and differential analysis. The mean entropy was approx. 7.9 and the mean number of pixels changing rate (NPCR) was approx. 99.8, unified average changing intensity (UACI) was approx. 33.46. These metrics indicate a high level of security and robustness in the encrypted images. The uniqueness of the performance analysis lies in its comprehensive, multi-dimensional evaluation approach that rigorously tests every critical aspect of the encryption algorithm. Unlike conventional studies that might focus on only one or two evaluation metrics, the analysis of this paper integrates a broad spectrum of tests.

- By assessing the randomness of the encrypted images the OptiSecure-3D model demonstrate the algorithm’s capability to produce highly diffused and secure ciphertext.
- Evaluating the PSNR between the original and decrypted images ensures that the encryption not only secures the data but also maintains the quality of the decrypted image.
- By applying randomness and statistical testing the OptiSecure-3D algorithm validate the effectiveness of the encryption in confusing original image patterns.
- Then pixel adjacency correlation test quantifies the reduction in correlation among adjacent pixels.
- The exceptionally high NPCR and optimal UACI values indicate strong key sensitivity.
- The performance under noise attacks confirms the robustness of the encryption scheme in real-world conditions where data transmission might be imperfect.

Despite these promising outcomes, the current work has certain limitations, such as potential computational overhead and challenges in adapting to more dynamic multimedia formats. In the future, this work will be extended to implement live-streaming videos.

## Data Availability

The datasets used and/or analysed during the current study available from the corresponding author on reasonable request.

## References

[1] Jasra, B. & Moon, A. H. Image encryption techniques: a review. In *10th International Conference on Cloud Computing, Data Science & Engineering (Confluence)*, Noida, India, 2020, pp. 221–226, (2020). 10.1109/Confluence47617.2020.9058071

[2] Chamoli, A., Ahmed, J., Alam, M. A. & Alankar, B. Analysis on optimal ways to secure image encryption and decryption. In *2nd International Conference on Advance Computing and Innovative Technologies in Engineering (ICACITE)*, Greater Noida, India, 2022, pp. 588–593, (2022). 10.1109/ICACITE53722.2022.9823565

[3] Kiya, H. Progress and challenges in compressible and learnable image encryption for privacy-preserving image encryption and machine learning [keynote]. In *2020 12th International Conference on Knowledge and Smart Technology (KST)*, Pattaya, Thailand, 2020, pp. XV-XV. 10.1109/KST48564.2020.9059423

[4] Abusukhon, A. & AlZu’bi, S. New direction of cryptography: A review on text-to-image encryption algorithms based on rgb color value. In *Seventh International Conference on Software Defined Systems (SDS)*, Paris, France, 2020, pp. 235–239, (2020). 10.1109/SDS49854.2020.9143891

[5] Xu, J., Ai, B., Chen, W., Yang, A. & Sun, P. Image encryption methods in deep joint source channel coding: A review and performance evaluation. In *2021 7th International Conference on Computer and Communications (ICCC)*, Chengdu, China, 2021, pp. 240–244. 10.1109/ICCC54389.2021.9674532

[6] Habek, M. et al. Digital image encryption using elliptic curve cryptography: A review,. In *2022 International Congress on Human-Computer Interaction, Optimization and Robotic Applications (HORA)*, Ankara, Turkey, 2022, pp. 1–8. 10.1109/HORA55278.2022.9800074

[7] Suneja, K., Dua, S., Dua, M., *Methodologies & Communication 3rd International Conference on Computing and A Review of Chaos based Image Encryption, (ICCMC)*, Erode, India, 2019, pp. 693–698, (2019). 10.1109/ICCMC.2019.8819860

[8] Paul, A. J. Recent advances in selective image encryption and its indispensability due to COVID-19. In *2020 IEEE Recent Advances in Intelligent Computational Systems (RAICS)*, Thiruvananthapuram, India, 2020, pp. 201–206. 10.1109/RAICS51191.2020.9332513

[9] Qayyum, A., Qadir, J., Bilal, M. & Al-Fuqaha, A. Secure and robust machine learning for healthcare: A survey. *IEEE Rev. Biomed. Eng.* **14**, 156–180. 10.1109/RBME.2020.3013489 (2021). 32746371 10.1109/RBME.2020.3013489

[10] Feng, W. et al. Cryptanalysis and improvement of the image encryption scheme based on feistel network and dynamic DNA encoding. *Ieee Access.* **9**, 145459–145470 (2021).

[11] Wen, H. & Lin, Y. Cryptanalyzing an image cipher using multiple chaos and DNA operations. *J. King Saud University-Computer Inform. Sci.* **35** (7), 101612 (2023).

[12] Akhtarkavan, E., Majidi, B. & Mandegari, A. Secure medical image communication using fragile data hiding based on discrete wavelet transform and a₅ lattice vector quantization. *IEEE Access* **11**, 9701–9715. 10.1109/ACCESS.2023.3238575 (2023).

[13] Lin, Y. et al. Blockchain-Aided secure semantic communication for AI-Generated content in metaverse. *IEEE Open. J. Comput. Soc.* **4**, 72–83. 10.1109/OJCS.2023.3260732 (2023).

[14] Xu, G., Li, G., Guo, S., Zhang, T. & Li, H. Secure decentralized image classification with multiparty homomorphic encryption. *IEEE Trans. Circuits Syst .Video Technol.* **33**(7), 3185–3198. 10.1109/TCSVT.2023.3234278 (2023).

[15] Fang, F., Liu, Y., Park, J. H. & Liu, Y. Outlier-resistant nonfragile control of T-S Fuzzy neural networks with reaction-diffusion terms and its application in image secure communication. *IEEE Trans Fuzzy Syst.* **31**(9), 2929–2942. 10.1109/TFUZZ.2023.3239732 (2023).

[16] He, W. et al. Secure communication based on quantized synchronization of chaotic neural networks under an event-triggered strategy. *IEEE Trans Neural Netw Learn Syst* **31**(9), 3334–3345. 10.1109/TNNLS.2019.2943548 (2020). 31634849 10.1109/TNNLS.2019.2943548

[17] Lv, Z., Chen, D., Cao, B., Song, H. & Lv, H. Secure deep learning in defense in deep-learning-as-a-service computing systems in digital twins. *IEEE Trans. Comput*. https://doi.org/10.1109/TC.2021.3077687.

[18] Choi, J. & Yu, N. Y. Secure image encryption based on compressed sensing and scrambling for internet-of-multimedia things. *IEEE Access* **10**, 10706–10718. 10.1109/ACCESS.2022.3145005 (2022).

[19] Boussif, M. & Mnassri, A. Secure images transmission using a three-dimensional s-box-based encryption algorithm. In *2022 5th International Conference on Advanced Systems and Emergent Technologies (IC_ASET)*, Hammamet, Tunisia, pp. 17–22, (2022). 10.1109/IC_ASET53395.2022.9765904

[20] Sarvepalli, G. P., Rethinam, S., Prasad, B. N. S. & Kulkarni, M. Secure image communication using systematic-LT codes over AWGN channel. In *2022 2nd Asian Conference on Innovation in Technology (ASIANCON)*, Ravet, India, 2022, pp. 1–7. 10.1109/ASIANCON55314.2022.9909507

[21] Ge, B. et al. Secure and fast image encryption algorithm using hyper-chaos-based key generator and vector operation. *IEEE Access.* **9**, 137635–137654 (2021).

[22] Kaur, M. et al. Lightweight biomedical image encryption approach. *IEEE Access.* **9**, 61334–61345 (2023).

[23] Qian, X. et al. A novel color image encryption algorithm based on three-dimensional chaotic maps and reconstruction techniques. *IEEE Access.* **9**, 61334–61345 (2021).

[24] Wen, H., Huang, Y. & Lin, Y. High-quality color image compression-encryption using chaos and block permutation. *J. King Saud University-Computer Inform. Sci.* **35**, 101660 (2023).

[25] Yuan, Y. et al. JPEG image encryption with grouping coefficients based on entropy coding. *J. Vis. Commun. Image Represent.* **97**, 103975 (2023).

[26] Lai, Q. et al. Image encryption using fission diffusion process and a new hyperchaotic map. Chaos. *Solitons Fractals*. **175**, 114022 (2023).

[27] Wen, H. & Lin, Y. Cryptanalysis of an image encryption algorithm using quantum chaotic map and DNA coding. *Expert Syst. Appl.* **237**, 121514 (2024).

[28] Guan, Q. et al. Multi-images encryption and watermarking with small number of keys via computational ghost imaging. *Opt. Laser Technol.* **168**, 109957 (2024).

[29] An, X. et al. Mixed gray-color images encryption algorithm based on a memristor chaotic system and 2D compression sensing. *Expert Syst. Appl.* **122899**. (2023).

[30] Kaur, M., Kumar, V. & Li, L. Color image encryption approach based on memetic differential evolution. *Neural Comput. Appl.* **31**, 7975–7987 (2019).

[31] Kaur, M., Singh, D. & Uppal, R. S. Parallel strength Pareto evolutionary algorithm-II based image encryption. *IET Image Process.* **14**(6), 1015–1026 (2020).

[32] Toktas, A., Erkan, U. & Ustun, D. ‘‘An image encryption scheme based on an optimal chaotic map derived by multi-objective optimization using ABC algorithm. *Nonlinear Dyn.* **105**(2), 1885–1909 (2021).

[33] Wang, J., Song, X. & Ahmed, A. Abd El-Latif. Single-objective particle swarm optimization-based chaotic image encryption scheme. *Electronics* **11**, 2628 (2022).

[34] Zhu, Y. et al. A chaotic image encryption method based on the artificial fish swarms algorithm and the DNA coding. *Mathematics* **11** (3), 767 (2023).

[35] Ma, X., Wang, Z. & Wang, C. An image encryption algorithm based on Tabu search and Hyperchaos. *Int. J. Bifurcat. Chaos*. **34** (14), 2450170 (2024).

[36] Deng, Q. et al. Memristive Tabu learning neuron generated multi-wing attractor with FPGA implementation and application in encryption. *IEEE Trans. Circuits Syst. I Regul. Pap.* **72** (1) 300–311 (2024).

[37] Chen, Y. et al. A novel adaptive image privacy protection method based on Latin square. *Nonlinear Dyn.* **112** (12), 10485–10508 (2024).

[38] Feng, W. et al. A novel multi-channel image encryption algorithm leveraging pixel reorganization and hyperchaotic maps. *Mathematics* **12**(24), 3917 (2024).

[39] Feng, W. et al. Exploiting robust quadratic polynomial hyperchaotic map and pixel fusion strategy for efficient image encryption. *Expert Syst. Appl.* **246**, 123190 (2024).

[40] Feng, W. et al. Exploiting newly designed fractional-order 3D Lorenz chaotic system and 2D discrete polynomial hyper-chaotic map for high-performance multi-image encryption. *Fractal Fract.* **7** (12), 887 (2023).

[41] Hua, Z., Yi, S. & Zhou, Y. Medical image encryption using high-speed scrambling and pixel adaptive diffusion. *Sig. Process.* **144**, 134–144 (2018).

[42] Sheng, Y. et al. An image encryption algorithm based on complex network scrambling and multi-directional diffusion. *Entropy* **24**(9), 1247 (2022). 36141133 10.3390/e24091247 PMC9498115

[43] Fridrich, J. Image encryption based on chaotic maps. In *IEEE International Conference on Systems, Man, and Cybernetics. Computational Cybernetics and Simulation.* vol. 2. pp. 1105–1110 (1997).

[44] Liu, Q. et al. A novel image encryption algorithm based on chaos maps with Markov properties. *Commun. Nonlinear Sci. Numer. Simul.* **20** (2), 506–515 (2015).

[45] Lorenz, E. N. Deterministic nonperiodic flow. *J. Atmos. Sci.* **20** (2), 130–141 (1963).

[46] Ali, N. A., Rahma, A. M. S. & Shaker, S. H. Multi-level encryption for 3D mesh model based on 3D Lorenz chaotic map and random number generator. *Int. J. Electr. Comput. Eng.* **12**(6), 8708 (2022).

[47] Chen, G. & Mao, Y. Chui. A symmetric image encryption scheme based on 3D chaotic Cat maps. *Chaos Solitons Fractals*. **21** (3), 749–761 (2004).

[48] Ding, Y. et al. DeepEDN: A deep-learning-based image encryption and decryption network for internet of medical things. *IEEE Internet Things J.* **8** (3), 1504–1518 (2020).

[49] Guan, Z. H., Huang, F. & Guan, W. Chaos-based image encryption algorithm. *Phys. Lett. A. ***346**, 153–157 (2005).

[50] Diaconu, A. V., & Dascalescu, A.C. Correlation distribution of adjacent pixels randomness test for image encryption. *Proc. Rom. Acad. Ser. A.* **18** (2017).

[51] Li, C. et al. Cryptanalysis of a chaotic image encryption algorithm based on information entropy. *Ieee Access.* **6**, 75834–75842 (2018).

[52] Ding, D. et al. An n-dimensional modulo chaotic system with expected Lyapunov exponents and its application in image encryption. *Chaos Solitons Fractals*. **174**, 113841 (2023).

[53] Wu, Y. & Noonan, J. P. NPCR and UACI randomness tests for image encryption. Cyber journals: multidisciplinary journals in science and technology. *J. Sel. Areas Telecommunications (JSAT)*. **1** (2), 31–38 (2011).

[54] Li, L. A novel chaotic map application in image encryption algorithm. *Expert Syst. Appl.* **124316** (2024).

[55] Liang, C. & Zhu A new one-dimensional chaotic map for image encryption scheme based on random DNA coding. *Opt. Laser Technol.* **160**, 109033 (2023).

[56] Zhang, Y., Chen, A., Tang, Y., Dang, J. & Wang, G. Plaintext-related image encryption algorithm based on perceptron-like network. *Inf. Sci.* **526**, 180–202 (2020).

[57] Kanwal, S. & Ali, R. A cryptosystem with noncommutative platform groups. *Neural Comput. Appl.* **29**, 1273–1278 (2018).

[58] Kanwal, S. et al. A new image encryption technique based on sine map, chaotic tent map, and circulant matrices. *Secur. Commun. Netw*. **4152683** (2022).

[59] Inam, S. et al. A new method of image encryption using advanced encryption standard (AES) for network security. *Phys. Scr.* **98** (12), 126005 (2023).

[60] Kanwal, S. et al. An efficient image encryption algorithm using 3D-cyclic Chebyshev map and elliptic curve. *Sci. Rep.* **14** (1), 29626 (2024). 39609493 10.1038/s41598-024-77955-w PMC11605050

[61] https://figshare.com/articles/dataset/BSD100_Set5_Set14_Urban100/21586188

[62] https://www.kaggle.com/datasets/navoneel/brain-mri-images-for-brain-tumor-detection

[63] Alghamdi, Y. & Munir, A. Image encryption algorithms: a survey of design and evaluation metrics. *J. Cybersecur. Priv.* **4** (1), 126–152 (2024).

